# Reimagining A Caregiver-friendly Society

**DOI:** 10.13023/jah.0503.01

**Published:** 2023-12-01

**Authors:** Jodi L. Southerland

**Affiliations:** East Tennessee State University, southerlanjl@etsu.edu

**Keywords:** Appalachia, aging, caregiving, policy, social policy

## Abstract

Demographic aging is accelerating in the Appalachian Region, resulting in a growing proportion of caregivers living in areas that lack services to support their needs. Strategies are urgently needed in Appalachia to address deficiencies in the region’s long-term supports and services for older adults and their caregivers. Strengthening equitable access to care and community supports for family caregivers is a policy priority for state and community leaders in Appalachia.

## INTRODUCTION

Approximately 53 million Americans (one in five) are family caregivers.^1^–^3^ These individuals provide roughly 35 billion hours of unpaid care annually, which account for over 75% of the caregiving provided in the U.S.^4^ Based on conservative estimates, the annual cost of this care ranges from $470 to $522 billion.^5^–^7^ There is increasing attention on caregiving as a social determinant of health, which is an important consideration in Appalachia.^8^ The Appalachian Region is disproportionately burdened with poor social, health, and economic outcomes.^9^ Yet a recent study found that older adults in rural Appalachia face more structural barriers to accessing home and community-based services than their urban counterparts.^10^ Additionally, caregivers in rural Appalachia often lack access to health and social services, have limited financial resources, and are at heightened risk for food insecurity.^9^–^11^ These factors contribute to poor self-care behaviors, which are associated with the early onset and progression of chronic conditions such as cardiovascular disease.^11^

Although caregiving is gratifying, the physical, emotional, and financial costs are well documented.^8^,^11^–^18^ Forty percent of caregivers are in a high-burden caregiving situation^7^ and three-fourths of working caregivers report significant job-related challenges affecting performance, longevity, and workplace well-being. 14,15 Caregivers spend as much as a fifth of their income on caregiving activities. Racial and ethnic minorities, individuals assisting with at least one activity of daily living, and individuals caring for someone with a dementia-related disease are disproportionately financially burdened.^16^,^17^

Research on population forecasting shows that the need for family caregivers will continue to grow with the acceleration of demographic aging.^19^–^21^ The older adult population is projected to double between 2000 and 2060. The number of individuals 85 and older, the fastest growing segment of the population, and those most in need of care will triple during this period.^22^ Increased longevity is a remarkable accomplishment of modern medical and public health achievements, but it has exposed deficiencies in our infrastructure and systems.^1^ For example, the proportion of older adults in the Appalachian Region is accelerating, resulting in a growing proportion of caregivers living in areas that lack services to support their needs.^23^–^29^ Additionally, over three-fourths of older Americans have at least two chronic age-related diseases,^27^ and the majority receive care assistance from a family member.^30^ These factors place a significant burden on caregivers in Appalachia.

Although caregivers are a cornerstone of society and support the needs of millions of older Americans, they rarely receive formal training before embarking on their caregiving journey.^7^,^31^ Lack of training contributes to excess burden of caregiving, resulting in compromised health.^7^,^32^ Eventually, the caregiver will become the patient and need care themselves. How can society support caregivers to ensure they have the resources to maintain their health while caring for our aging population?^33^

The roadmap to healthy longevity begins with national and state-level policy reform.

The 2022 National Strategy to Support Family Caregivers represents a significant step forward in our progress toward comprehensive reform of current long-term supports and services (LTSS), caregiver compensation, and workplace and family medical leave policies.^33^–^35^ Financing for community-based LTSS and paid family caregiver leave are two of the most crucial strategies needed.^36^ States play a critical role and are being called upon to develop robust plans to support caregivers: policy strategies, surveillance, expanded services, and mechanisms to systematically evaluate promising practices^37^ to ensure quality aging for everyone.^38^ Strategies are urgently needed in Appalachia to address deficiencies in the region’s LTSS system for older adults and their caregivers.^39^ Strengthening equitable access to care and community supports for family caregivers must be a policy priority for state and community leaders in Appalachia.

Caregiving touches all our lives, whether we realize it or not. In the words of former First Lady Rosalyn Carter, “There are only four kinds of people in the world: those who have been caregivers, those who are currently caregivers, those who will be caregivers, and those who will need a caregiver.”^8^ We can reimagine a more compassionate and supportive society by centering the needs of older persons and family caregivers in policy-making decisions. Investing in an age-friendly and caregiver-friendly society^40^ is an investment for all.
